# Diagnostic Accuracy of Urine Protein/Creatinine Ratio Is Influenced by Urine Concentration

**DOI:** 10.1371/journal.pone.0137460

**Published:** 2015-09-09

**Authors:** Chih-Yu Yang, Fu-An Chen, Chun-Fan Chen, Wen-Sheng Liu, Chia-Jen Shih, Shuo-Ming Ou, Wu-Chang Yang, Chih-Ching Lin, An-Hang Yang

**Affiliations:** 1 Division of Nephrology, Department of Medicine, Taipei Veterans General Hospital, Taipei, Taiwan; 2 Department of Pathology and Laboratory Medicine, Taipei Veterans General Hospital, Taipei, Taiwan; 3 Institute of Clinical Medicine, National Yang-Ming University, Taipei, Taiwan; 4 School of Medicine, National Yang-Ming University, Taipei, Taiwan; 5 Division of Nephrology, Department of Medicine, National Yang-Ming University Hospital, Yilan, Taiwan; 6 Division of Nephrology, Department of Medicine, Taipei City Hospital Zhongxing Branch, Taipei, Taiwan; 7 School of Medicine, Fu Jen Catholic University, New Taipei City, Taiwan; 8 Division of Nephrology, Department of Medicine, Taipei Veterans General Hospital Yuanshan Branch, Yilan, Taiwan; Cedars-Sinai Medical Center, UNITED STATES

## Abstract

**Background:**

The usage of urine protein/creatinine ratio to estimate daily urine protein excretion is prevalent, but relatively little attention has been paid to the influence of urine concentration and its impact on test accuracy. We took advantage of 24-hour urine collection to examine both urine protein/creatinine ratio (UPCR) and daily urine protein excretion, with the latter as the reference standard. Specific gravity from a concomitant urinalysis of the same urine sample was used to indicate the urine concentration.

**Methods:**

During 2010 to 2014, there were 540 adequately collected 24h urine samples with protein concentration, creatinine concentration, total volume, and a concomitant urinalysis of the same sample. Variables associated with an accurate UPCR estimation were determined by multivariate linear regression analysis. Receiver operating characteristic (ROC) curves were generated to determine the discriminant cut-off values of urine creatinine concentration for predicting an accurate UPCR estimation in either dilute or concentrated urine samples.

**Results:**

Our findings indicated that for dilute urine, as indicated by a low urine specific gravity, UPCR is more likely to overestimate the actual daily urine protein excretion. On the contrary, UPCR of concentrated urine is more likely to result in an underestimation. By ROC curve analysis, the best cut-off value of urine creatinine concentration for predicting overestimation by UPCR of dilute urine (specific gravity ≦ 1.005) was ≦ 38.8 mg/dL, whereas the best cut-off values of urine creatinine for predicting underestimation by UPCR of thick urine were ≧ 63.6 mg/dL (specific gravity ≧ 1.015), ≧ 62.1 mg/dL (specific gravity ≧ 1.020), ≧ 61.5 mg/dL (specific gravity ≧ 1.025), respectively. We also compared distribution patterns of urine creatinine concentration of 24h urine cohort with a concurrent spot urine cohort and found that the underestimation might be more profound in single voided samples.

**Conclusions:**

The UPCR in samples with low or high specific gravity is more likely to overestimate or underestimate actual daily urine protein amount, respectively, especially in a dilute urine sample with its creatinine below 38.8 mg/dL or a concentrated sample with its creatinine above 61.5 mg/dL. In particular, UPCR results should be interpreted with caution in cases that involve dilute urine samples because its overestimation may lead to an erroneous diagnosis of proteinuric renal disease or an incorrect staging of chronic kidney disease.

## Introduction

The diagnosis and management of proteinuric renal diseases and the staging of chronic kidney disease (CKD) require accurate identification and quantitation of proteinuria [1]. Utilization of 24-hour (24h) urine collection is considered the gold standard with regards to methods that determine urinary protein excretion. However, in current clinical practice, spot urine protein/creatinine ratio (UPCR) is widely used to estimate daily protein excretion by virtue of its convenience and simplicity [2]. The Kidney Disease Outcomes Quality Initiative (K/DOQI) of the National Kidney Foundation Practice Guideline recommended the use of “spot” urine protein/creatinine measurements to detect proteinuria when staging CKD; it recommended that under most circumstances, untimed (“spot”) urine sample should be used to detect and monitor proteinuria in children and adults and it is usually not necessary to obtain a timed urine collection (overnight or 24-hour) for these evaluations in either children or adults [1;3;4]. In addition, the American Diabetes Association (ADA) also strongly encouraged a spot urine sample for the quantitative albuminuria or proteinuria, whereas 24h collection or a timed specimen are rarely necessary while screening for microalbuminuria or proteinuria in diabetic patients [5–7]. Nevertheless, it’s unknown whether urine concentration affects the accuracy of UPCR estimation.

The concept of UPCR is to use urine creatinine to eliminate the effect of concentration status of urine. Although urine creatinine is positively correlated with urine specific gravity [8;9], it is also affected by muscle mass, animal protein intake, strenuous exercise, or certain drug usage when compared to specific gravity [10–12]. Moreover, one can simply reason that urine creatinine concentration may become very low in an extreme dilute urine sample. Taking a dilute urine sample with its protein concentration as 3.0 mg/dL and its creatinine concentration as 9.0 mg/dL for example, the estimated value will be 0.333 g/day. Meanwhile, for another example of a dilute urine sample with its protein concentration as 2.0 mg/dL and its creatinine concentration as 10.0 mg/dL, the estimated value will be 0.200 g/day. Although there is only 1 mg/dL difference in urine protein and creatinine concentrations, the results were quite different. This might potentially result in a wrong diagnosis of proteinuria or even a mistaken CKD staging, which relies on the presence of proteinuria as a biomarker of kidney damage [1]. Nevertheless, the influence of urine concentration on the accuracy of UPCR has not been systematically investigated.

We hypothesized that the status of urine concentration influences the accuracy of proteinuria quantitation estimated by UPCR. Urine specific gravity was used to indicate urine concentration. We used 24h urine protein excretion as the standard value, and the relationship between urine concentration and the accuracy of UPCR estimation was examined.

## Materials and Methods

### Ethics statement

The protocol of this study was approved by the Institutional Review Board of Taipei Veterans General Hospital, Taipei, Taiwan. The protocol conformed with the ethical guidelines of the *Helsinki Declaration*. The need for informed consent was waived because of the retrospective nature of the study.

### Study protocol and subjects

**Fig 1:**
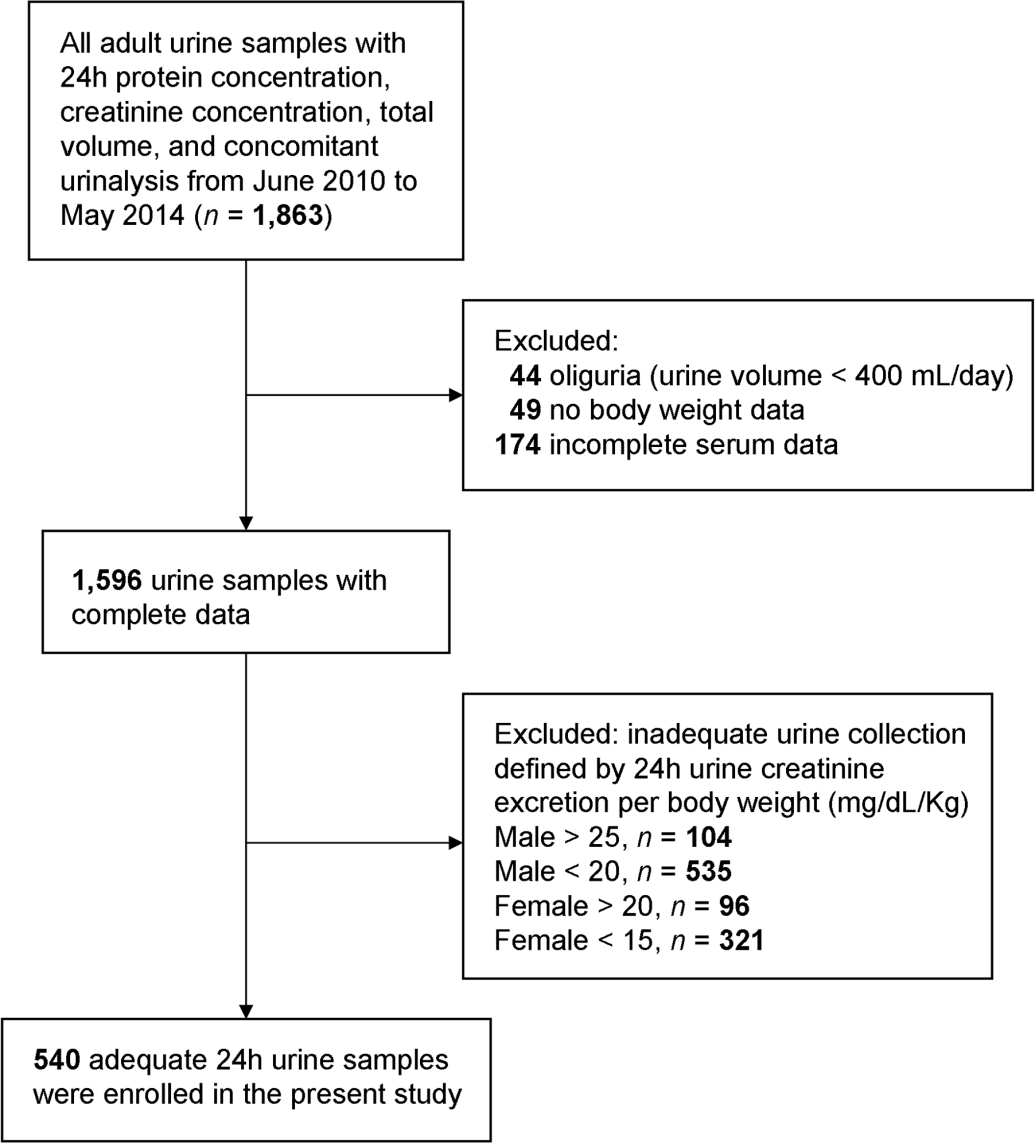
Flow diagram for the study.

We retrospectively collected the urine data obtained from June 2010 to May 2014 at the Taipei Veterans General Hospital, a tertiary-care referral hospital. We collected urine samples with complete data of 24h urine protein concentration, creatinine concentration, total volume, and most importantly, a concomitant urinalysis of the same urine sample. The 24h urine samples were collected either at home or at ward, and either self-voided or through a Foley catheter. The 24h urine samples were transferred from a clean collecting container to a clean storage container where it was kept in a cool environment. After the total volume of the 24h urine sample had been measured and the storage container had been gently shaken for 10 seconds to mix the urine, two 10 mL specimens were sent for urinalysis and urine protein creatinine concentration measurement, respectively. The urine protein and creatinine concentration of both 24h and spot urine were both determined by the Hitachi 7180 Autoanalyzer (Hitachi, Tokyo, Japan). The specific gravity of the urinalysis was analyzed by using the Clinitek Atlas Automated Urine Chemistry Analyzer (Siemens Healthcare Diagnostics, Erlangen, Germany). All patients were at least 18 years of age. As shown in Fig 1, we excluded oliguria defined by 24h urine volume less than 400 mL (*n* = 44), no body weight data (*n* = 49), incomplete serum data (*n* = 174), and inadequate urine collection defined by inadequate 24h urinary creatinine excretion per body weight (*n* = 1,056) [13]. Eventually, a total of 540 adequate 24h urine samples were enrolled. Meanwhile, in order to a compare distribution patterns of urine creatinine concentration between 24h and spot urine samples, a concurrent database of single voided urine sample during June 2010 to May 2014 was examined. Spot urine samples were collected at any time point of the day. There were 31,551 spot UPCR samples with a concomitant urinalysis examination.

The demographic features and clinical parameters, including age, gender, body mass index, comorbidities, serum biochemical data, 24h urine protein, creatinine, total volume, and concomitant urinalysis of the same sample were recorded. Serum laboratory data were obtained at the time point of urine collection. All measurements were determined in a single central laboratory. Estimated glomerular filtration rate (eGFR) was calculated by the Cockcroft-Gault formula [14]. UPCR was calculated from urine protein (mg/dL) divided by urine creatinine (mg/dL). 24h urine protein excretion was used as the standard reference, which was calculated by urine protein (mg/dL) multiplied by 24h urine volume (mL) and then converted and presented in gram/day. The percentage discrepancy between UPCR and 24h urine protein excretion (24h-UP) was calculated as (UPCR—24h-UP) ÷ 24h-UP × 100% to measure the diagnostic accuracy of UPCR.

### Statistical analysis

Values of the continuous variables are presented as mean and standard deviation or median and interquartile range, unless otherwise specified. Continuous variables were compared by analysis of variance (ANOVA) as appropriate. For linear regression analysis, the percentage discrepancy between UPCR and 24h-UP [(UPCR—24h-UP) ÷ 24h-UP × 100%] was used as the dependent variable, and serum and urine data including urine specific gravity (last two digits of the reported specific gravity) were used as independent variables. Variables associated with the dependent variable in univariate linear regression analysis with *p* value less than 0.10 were included in the multivariate linear regression analysis. Pearson’s correlation coefficient was used to evaluate bivariate relationships between urine specific gravity and creatinine concentration. Receiver operating characteristic (ROC) curve analyses were performed to determine the best cut-off values for predicting an overestimation of daily protein excretion by UPCR in dilute urine samples with specific gravity ≦ 1.005, and an underestimation in those with specific gravity ≧ 1.015, ≧ 1.020, and ≧ 1.025, respectively. To compare the distribution of urine creatinine concentration between 24h urine and spot urine cohorts for each particular specific gravity, histograms were plotted to indicate the percentage of frequency for every 10 mg/dL range of urine creatinine concentration and then intraclass correlation coefficients (Ri) between two urine cohorts were examined. SPSS version 15.0 for Windows (SPSS Inc., Chicago, Illinois, USA) was used for all statistical analyses. All probabilities were two-tailed, and a *p* value of less than 0.05 was considered to be statistically significant.

## Results

### Demographic characteristics of the study subjects

Table 1 shows the demographic characteristics of the 540 samples. The mean age was 53.1 years, 40.2% of the patients were male, and the body mass index was 23.8 ± 4.0. Sixteen point one percent had diabetes mellitus, 31.3% had hypertension, 8.0% had coronary artery disease, 6.3% had congestive heart failure, and 6.1% had systemic lupus erythematosus. The median 24h urine protein concentration was 18.6 mg/dL, median 24h urine creatinine concentration was 54.2 mg/dL, median total urine volume was 2110 mL, median 24h urine protein excretion was 0.437 g/day, and median UPCR was 0.348.

**Table 1:** Demographic characteristics and clinical data.

Characteristic	Value
Sample number (*n*)	540
Age (year)	53.1 ± 17.0
Male gender (%)	40.2
Body mass index (Kg/m^2^)	23.8 ± 4.0
**Comorbidities**	
Diabetes mellitus (%)	16.1
Hypertension (%)	31.3
Coronary artery disease (%)	8.0
Congestive heart failure (%)	6.3
Systemic lupus erythematosus (%)	6.1
**Serum**	
Albumin (g/dL)	4.0 ± 0.6
Total cholesterol (mg/dL)	199.7 ± 55.3
Blood urea nitrogen (mg/dL)	22.6 ± 16.6
Uric acid (mg/dL)	6.3 ± 1.8
Creatinine (mg/dL)	1.4 ± 1.3
eGFR (mL/min)	71.8 ± 43.3
**24h urine**	
Protein (mg/dL)	18.6 (7.0–66.0)
Creatinine (mg/dL)	54.2 (40.8–74.2)
Volume (mL)	2110 (1741–2700)
Daily urine protein excretion (g/24h)	0.437 (0.155–1.442)
Urine protein/creatinine ratio	0.348 (0.136–1.191)

Unless otherwise noted, values are expressed as mean ± standard deviation, percentage, or median (interquartile range). Abbreviation: eGFR, estimated glomerular filtration rate (Cockcroft-Gault formula).

### Univariate and multivariate linear regression analysis

In Table 2, we examined the variables associated with the percentage discrepancy between UPCR and 24h urine protein excretion by using both univariate and multivariate linear regression analysis. We found that serum uric acid (β = -1.765, *p* < 0.001), 24h urine creatinine concentration (β = -0.945, *p* < 0.001), 24h total urine volume (β = -0.027, *p* < 0.001), and specific gravity (β = -0.393, *p* = 0.002) were independently associated with the percentage discrepancy between UPCR and 24h urine protein excretion.

**Table 2:** Linear regression analysis of variables associated with the percentage discrepancy between urine protein/creatinine ratio and 24h urine protein excretion.

Variable	Univariate				Multivariate			
	β Coefficient	95% CI		*p* Value	β Coefficient	95% CI		*p* Value
		Lower	Upper			Lower	Upper	
					R^2^ = 0.699			
**Serum**								
Albumin (g/dL)	-0.809	-4.495	2.877	0.666				
Total cholesterol (mg/dL)	-0.023	-0.067	0.020	0.293				
Blood urea nitrogen (mg/dL)	-0.051	-0.183	0.082	0.451				
Uric acid (mg/dL)	-4.093	-5.370	-2.817	<0.001*	-1.765	-2.604	-0.926	<0.001*
eGFR (mL/min)	-0.172	-0.221	-0.122	<0.001*	-0.002	-0.039	0.035	0.905
**24h urine**								
Protein (mg/dL)	-0.017	-0.030	-0.004	0.009*	0.022	-0.024	0.068	0.351
Creatinine (mg/dL)	-0.509	-0.573	-0.444	<0.001*	-0.945	-1.020	-0.870	<0.001*
Volume (mL)	-0.003	-0.006	0.001	0.067	-0.027	-0.030	-0.024	<0.001*
24h urine protein excretion (g/24h)	-1.095	-1.866	-0.324	0.005*	-1.291	-3.524	0.941	0.256
Urine protein/creatinine ratio	-0.218	-1.138	0.702	0.642				
Specific gravity (last two digits)	-1.241	-1.572	-0.910	<0.001*	-0.393	-0.634	-0.151	0.002*

Abbreviation: eGFR, estimated glomerular filtration rate (Cockcroft-Gault formula).

*p < 0.05.

### Urine specific gravity and the accuracy of UPCR estimation

**Fig 2:**
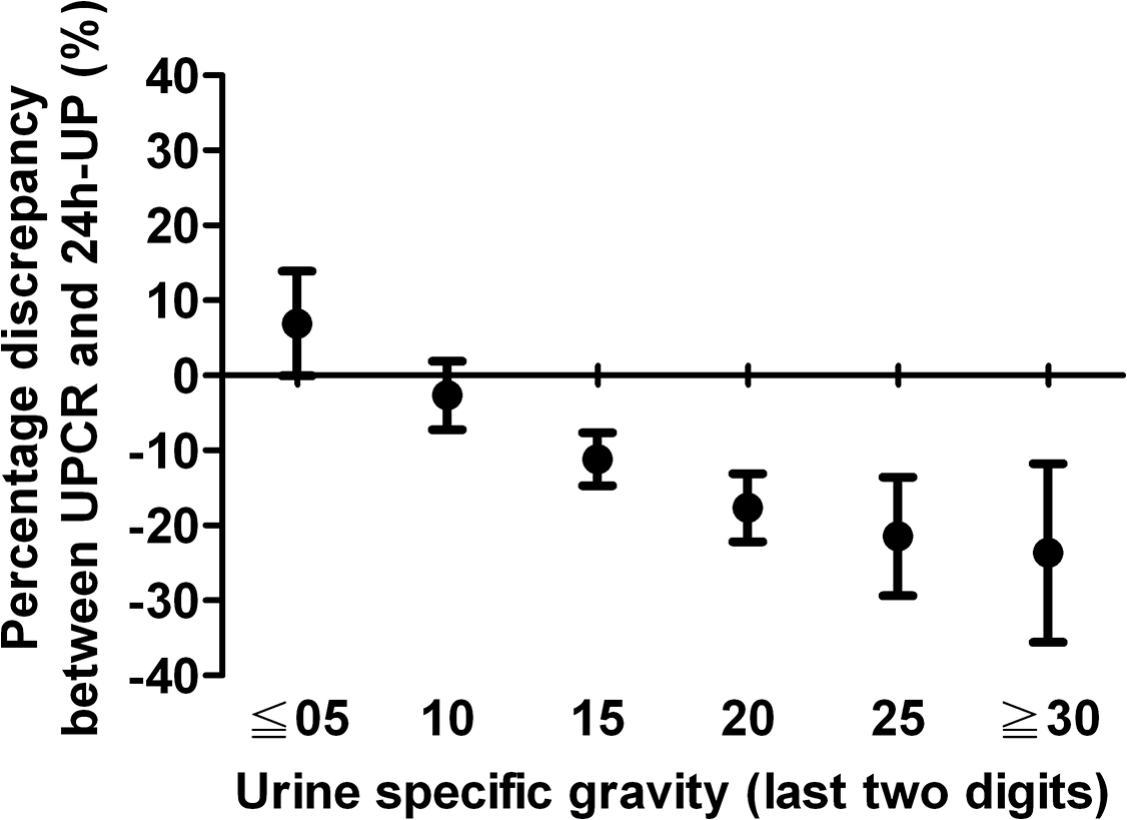
Association of urine specific gravity and the percentage discrepancy between urine protein/creatinine ratio (UPCR) and 24-hour urine protein excretion (24h-UP). for dilute urine, UPCR overestimates 24h-UP. As the urine specific gravity increases, UPCR underestimates 24h-UP. Dot: mean; bar: 95% confidence interval.

Because urine specific gravity was independently associated with the discrepancy between UPCR and 24h-UP, urine samples were categorized into 6 groups according to their specific gravity. Groups 1 to 6 were defined as follows, group 1, specific gravity ≦ 1.005 (*n* = 46); group 2, specific gravity = 1.010 (*n* = 123); group 3, specific gravity = 1.015 (*n* = 182); group 4, specific gravity = 1.020 (*n* = 132); group 5, specific gravity = 1.025 (*n* = 46), and group 6, specific gravity ≧ 1.030 (*n* = 11). As shown in Fig 2, for group 1 UPCR overestimates actual daily urine protein excretion, whereas for groups 3–6 the actual values were underestimated. The mean percentage of overestimation by UPCR in group 1 was 6.9%. The mean percentage of underestimation by UPCR in groups 3 to 6 were 11.2%, 17.7%, 21.5%, and 23.7%, respectively.

### Positive correlation between urine specific gravity and creatinine concentration

**Fig 3:**
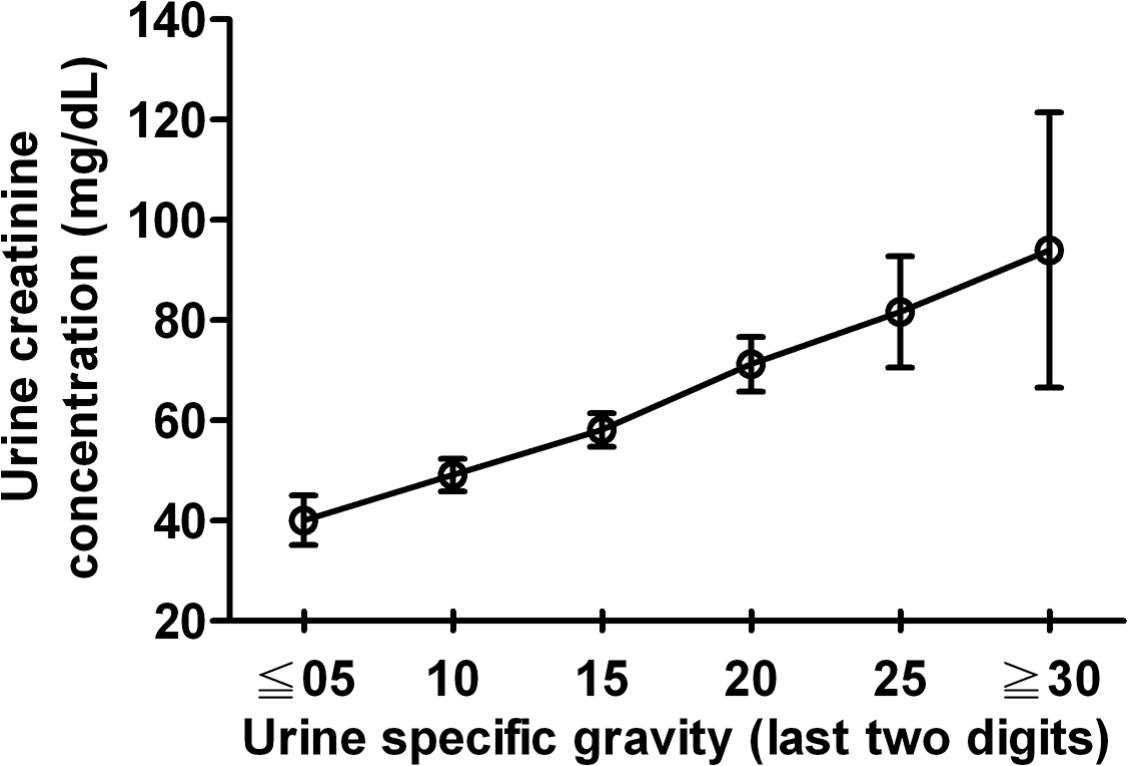
Relationship between urine specific gravity and creatinine concentration. Urine specific gravity is positively correlated with urine creatinine concentration. Dot: mean; bar: 95% confidence interval.

We tested the Pearson’s correlation between urine specific gravity and creatinine concentration. There is a positive correlation between them (r = 0.430; *p* < 0.001). Such a positive correlation was shown in Fig 3 and categorized by urine specific gravity.

### Sensitivity and specificity of urine creatinine concentration

**Fig 4:**
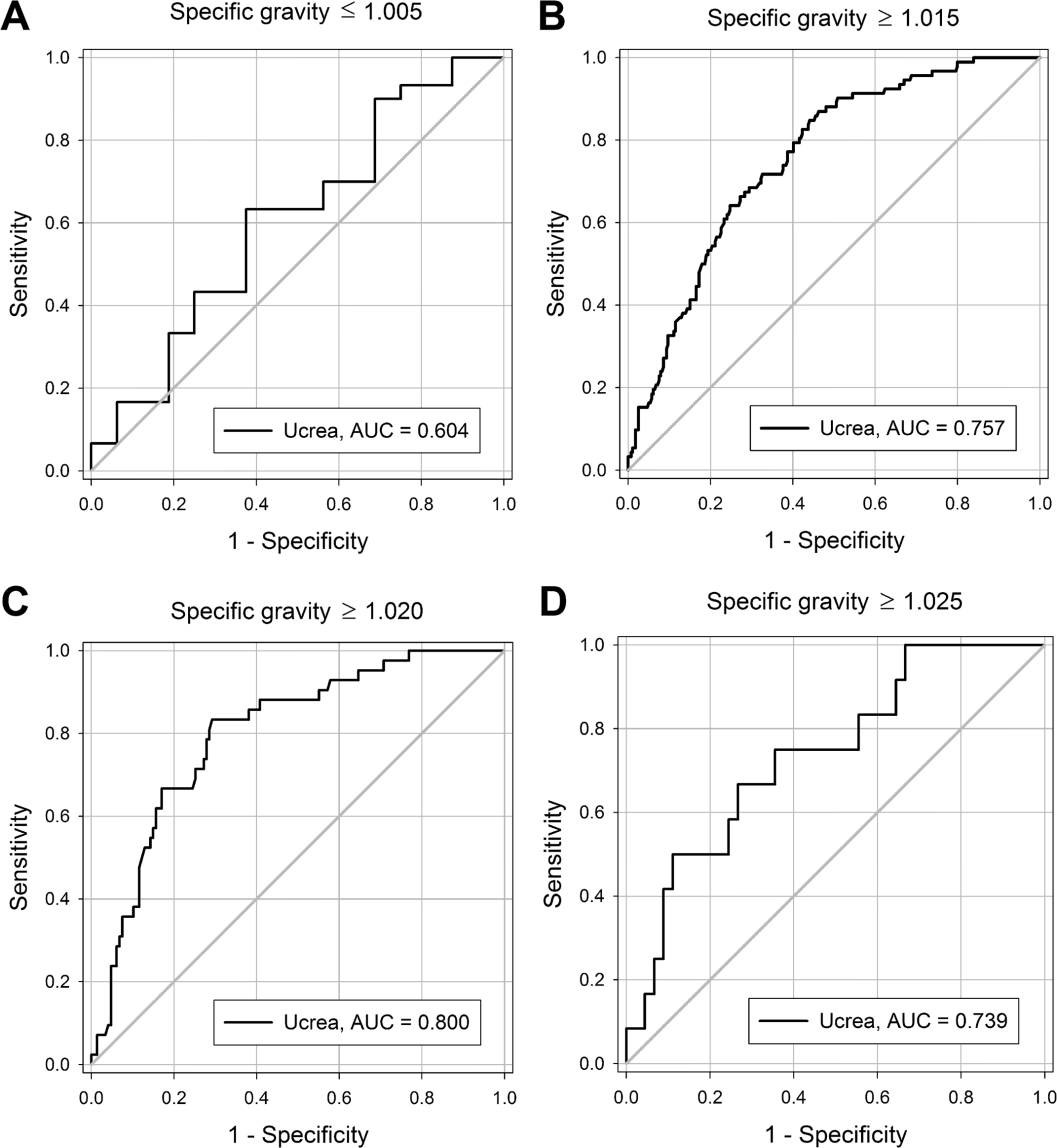
Receiver operating characteristic (ROC) curves of urine creatinine concentration (Ucrea; mg/dL) for predicting urine protein/creatinine ratio (UPCR) accuracy. ROC curves of urine creatinine concentration predicted overestimation of daily protein excretion by UPCR in urine samples with specific gravity ≦ 1.005 (panel A: specific gravity category group 1, *n* = 46; cut-off value result: Ucrea ≦ 38.8 mg/dL), and predicted underestimation in those specific gravity ≧ 1.015 (panel B: specific gravity category groups 3–6, *n* = 371; cut-off value result: Ucrea ≧ 63.6 mg/dL), in those specific gravity ≧ 1.020 (panel C: specific gravity category groups 4–6, *n* = 189; cut-off value result: Ucrea ≧ 62.1 mg/dL), and in those specific gravity ≧ 1.025 (panel D; specific gravity category groups 5–6, *n* = 57; cut-off value result: Ucrea ≧ 61.5 mg/dL), respectively. Abbreviation: AUC, area under curve.

Since UPCR reports are usually not accompanied by specific gravity from a concomitant urinalysis and there is a positive correlation between urine specific gravity and creatinine concentration, receiver operating characteristic (ROC) curves were constructed to determine the cut-off values of urine creatinine concentration for predicting an accurate UPCR estimation. We found that for dilute urine samples with specific gravity ≦ 1.005, a sample with urine creatinine ≦ 38.8 mg/dL was more likely to overestimate actual daily urine protein excretion by using UPCR (Fig 4A). On the other hand, for urine samples with specific gravity ≧ 1.015, ≧ 1.020, and ≧ 1.025, those with urine creatinine concentration ≧ 63.6 mg/dL, ≧ 62.1 mg/dL, and ≧ 61.5 mg/dL, respectively, were more likely to lead to an underestimation (Fig 4B–4D).

### Distribution patterns of urine creatinine concentration between 24h urine versus spot urine cohorts

**Fig 5:**
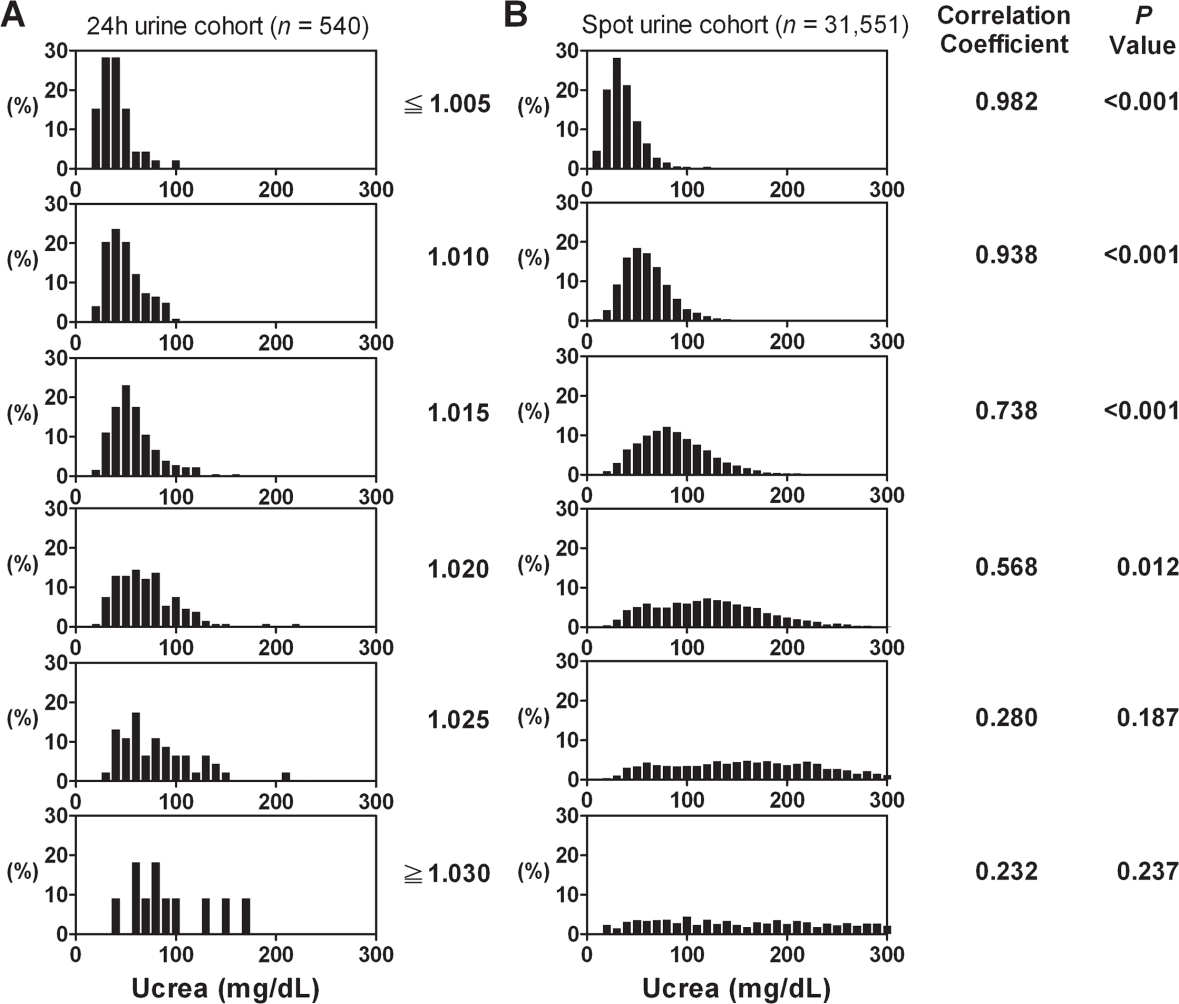
Histograms of urine creatinine concentration of the 24h urine cohort (A) and the spot urine cohort (B). Urine samples were categorized into 6 groups according to urine specific gravity. The Y-axis indicated the percentage of frequency in the particular category. The correlation between the two cohorts in each group was presented with intraclass correlation coefficients.

**Fig 6:**
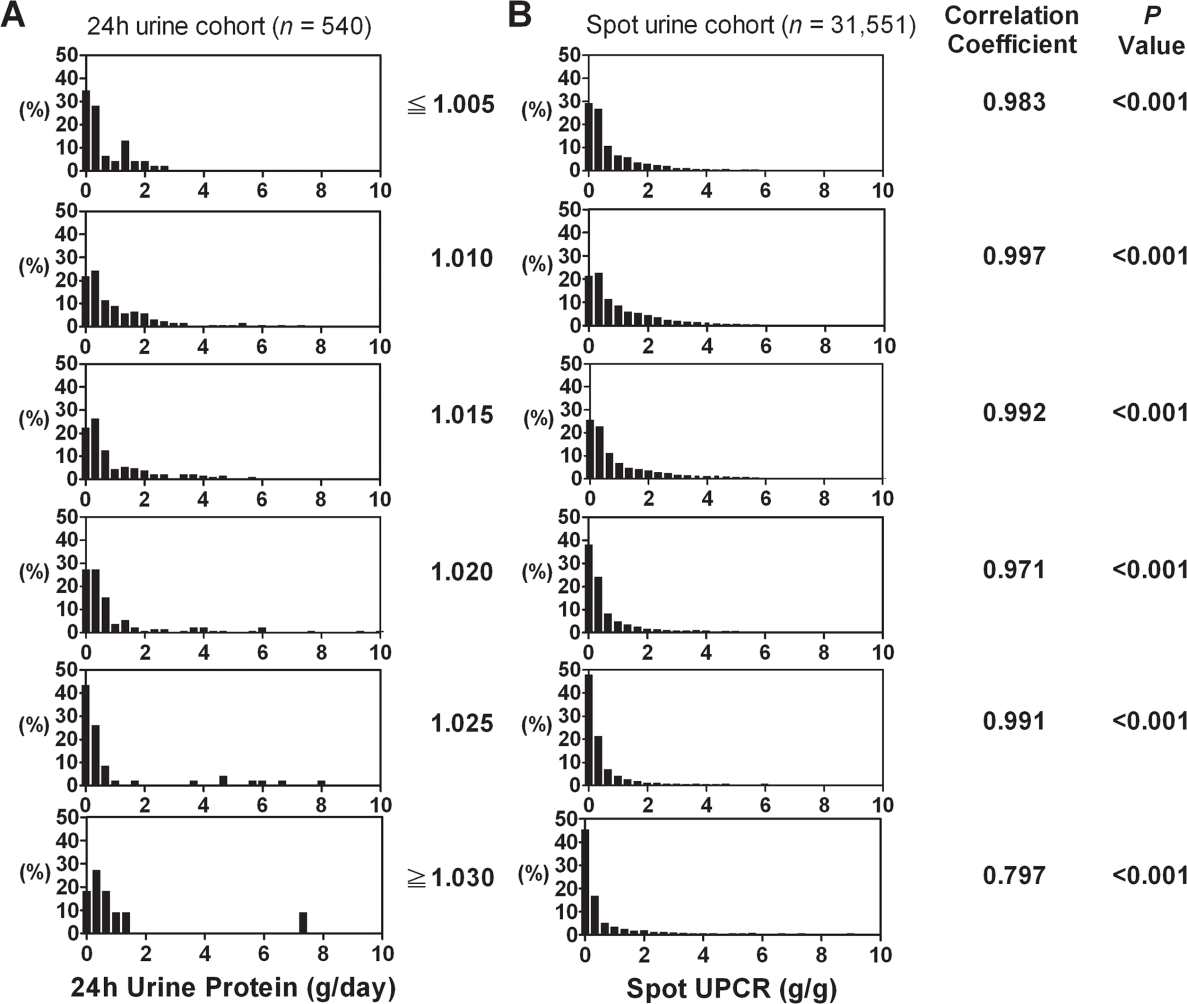
Histograms of quantitative daily protein excretion of the 24h urine cohort (A) and the spot urine cohort (B). Urine samples were categorized into 6 groups according to urine specific gravity. The Y-axis indicated the percentage of frequency in the particular category. The correlation between the two cohorts in each group was presented with intraclass correlation coefficients.

Given the fact that UPCR accuracy requires a 24h urine protein amount to serve as a reference standard, our results were derived from 24h adequate urine samples. However, whether our findings may apply to single voided urine samples is unknown. Therefore, we collected a spot urine cohort with a concomitant urinalysis of the same period (*n* = 31,551) to examine the distribution pattern of urine creatinine concentration. We found that the similarity of urine creatinine concentration between two cohorts were highest in dilute urine (urine specific gravity ≦ 1.005, Ri = 0.987, *p* < 0.001, Fig 5). As the urine specific gravity increased, the intraclass correlation coefficient declined but still achieved a significant correlation for urine specific gravity = 1.020 (Ri = 0.268, *p* = 0.012, Fig 5). As shown in Fig 5, for thick urine (urine specific gravity ≧ 1.025), urine creatinine concentration of the spot urine cohort was more widely distributed with the values as high as 300 mg/dL, which further increases the likelihood of urine protein underestimation when using UPCR. In addition, we further examined the correlation degree of quantitative daily urine protein excretion between the 24h and spot urine cohorts, which showed a statistically significant similarity throughout the 6 groups with different specific gravities (Ri = 0.983–0.797, *p* < 0.001, Fig 6).

## Discussion

Beyond the fact that the 24h collected urine specimen offers additional information to assess key dietary parameters and creatinine clearance, the 24h urine protein quantitation is more accurate than spot UPCR; however, it’s inconvenient for patients. In contrast, spot UPCR is the simplest method to quantitate proteinuria [1;15]. It has been reported that urine specific gravity may be used to normalize for the varied urine concentration while screening for microalbuminuria using a dipstick test [8;9;16]. However, the influence of urine concentration as indicated by specific gravity on the diagnostic accuracy of UPCR has not been investigated previously. In the present study, multivariate linear regression analysis revealed that urine specific gravity is independently associated with UPCR accuracy. Our study further demonstrated that for dilute urine, as indicated by a low urine specific gravity, UPCR is more likely to overestimate the actual daily urine protein amount. On the contrary, for concentrated urine the actual proteinuria values are more likely to be underestimated by UPCR.

Our urine samples were collected with a concomitant data of urine specific gravity, which was not available in UPCR reports in daily practice. Because our cohort and previous studies demonstrated that there is a positive correlation between urine specific gravity and creatinine concentration [8;9], a low urine creatinine can also be served as an indicator of dilute urine beyond specific gravity. Moreover, multivariate linear regression analysis showed that urine creatinine concentration is also independently associated with UPCR accuracy beyond specific gravity. Therefore, we further performed an ROC curve analysis to determine the discriminant cut-off values of urine creatinine concentration for predicting an accurate UPCR estimation. We found that the dilute urine sample with its creatinine concentration less than or equal to 38.8 mg/dL is more likely to overestimate actual urine protein excretion by UPCR. In clinical practice, spot urine sample with urine creatinine concentration less than 38.8 mg/dL is not infrequent. Therefore, interpretation of a spot UPCR report with an equivocal proteinuria result should be done so cautiously if the ratio was derived from a low urine creatinine, and a repeated examination or a timed urine sample collection may be indicated for a more precise confirmation. This is essential not only for an accurate diagnosis of proteinuria but also for a correct CKD staging, which relies on the presence of proteinuria as a biomarker of kidney damage.

The ROC curve analysis also demonstrated that the urine sample with a creatinine concentration more than or equal to 61.5 mg/dL is more likely to underestimate actual urine protein excretion by UPCR, according to subgroup analyses of urine samples with specific gravity ≧ 1.015, ≧ 1.020, or ≧ 1.025, respectively. Along with the aforementioned findings with regards to dilute urine, our findings suggest that a urine sample with its creatinine concentration within the range of 38.8 to 61.5 mg/dL is the least likely to either overestimate or underestimate the actual daily protein amount. Although both dilute and concentrated urine leads to inaccurate estimation by UPCR, the impact of overestimation in dilute urine is more profound in clinical practice because even a 10% overestimation by UPCR might lead to an inaccurate diagnosis of proteinuria.

We used 24h urine protein excretion as the diagnostic standard, and as such we had to confirm that the 24h urine samples were adequately collected. The completeness of a 24h urine collection is not assessed by volume but rather by urinary creatinine excretion [17]. The 24h urinary creatinine excretion reflects muscle mass, and its excretion is relatively constant over time in a given person [18]. Therefore, our findings were based on a database of adequately collected 24h urine samples, which was defined by urinary creatinine excretion (15 to 25 mg/Kg per day in men, and 15–20 mg/Kg per day in women) according to Walser *et al*. [13]. We further compared the distribution patterns of urine creatinine concentration between 24h urine and spot urine cohorts. The results showed that for dilute urine (urine specific gravity ≦ 1.005), there was no discrepancy between them (Fig 5). Therefore, the cut-off value of urine creatinine ≦ 38.8 mg/dL for predicting an UPCR overestimation in dilute urine should be applicable in spot urine samples.

On the other hand, for thick urine, the distribution of urine creatinine concentration in the spot urine cohort was not only wider but also showed a trend towards a higher value as compared to that of the present study (Fig 5). In other words, the average urine creatinine of spot urine was higher than that of a 24h sample with the same specific gravity, and higher urine creatinine levels should exaggerate the underestimation because urine creatinine is the denominator of UPCR formula. Therefore, the degree of underestimation should be more profound in concentrated spot urine sample than that of 24h sample with the same specific gravity, and the cut-off values of urine creatinine as 61.5 mg/dL to predict an underestimation by UPCR in thick urine should also be able to apply to single voided UPCR.

Our study was limited by its retrospective design and a relatively small scale of sample size. Nevertheless, our study unveiled this important yet previously unrecognized issue. Further larger scale or even nationwide studies are warranted. Another limitation is that our findings were based on a 24h urine sample database. However, we compared the distribution patterns of urine creatinine between 24h urine and spot urine cohorts, which showed a similar result in dilute urine and possibly an even more profound underestimation in concentrated spot urine samples. Finally, the spot urine cohort lacks the information of the timing of urine collection due to the retrospective nature of our study; however, the K/DOQI guideline recommended that random urine specimens are acceptable if first morning urine specimens are not available [1].

In conclusion, our study demonstrated the influence of urine concentration status on UPCR accuracy. In particular, our findings suggest that an equivocal proteinuria result derived from UPCR with a low urine creatinine concentration should be interpreted with caution because it might lead to an erroneous diagnosis of proteinuric renal disease or even an incorrect staging of CKD.
